# A Transfer Function Model Development for Reconstructing Radial Pulse Pressure Waveforms Using Non-Invasively Measured Pulses by a Robotic Tonometry System

**DOI:** 10.3390/s21206837

**Published:** 2021-10-14

**Authors:** Gwanghyun Jo, Tae-Heon Yang, Jeong-Hoi Koo, Min-Ho Jun, Young-Min Kim

**Affiliations:** 1 Department of Mathematics, Kunsan National University, 558 Daehak-ro, Gunsan-si 54150, Korea; gwanghyun@kunsan.ac.kr; 2 Department of Electronic Engineering, Korea National University of Transportation, 50 Daehak-ro, Chungju-si 27469, Korea; 3 Department of Mechanical and Manufacturing Engineering, Miami University, Oxford, OH 45242, USA; koo@miamioh.edu; 4 Digital Health Research Division, Korea Institute of Oriental Medicine, 1672, Yuseong-daero, Yuseong-gu, Daejeon 34054, Korea; mino@kiom.re.kr

**Keywords:** robotic tonometry system, tonometric measurement, radial transfer function, radial pulse pressure waveforms, pulse pressure generator

## Abstract

The primary goal of this study is to develop a mathematical model that can establish a transfer function relationship between the “external” pulse pressures measured by a tonometer and the “internal” pulse pressure in the artery. The purpose of the model is to accurately estimate and rebuild the internal pulse pressure waveforms using arterial tonometry measurements. To develop and validate a model without human subjects and operators for consistency, this study employs a radial pulse generation system, a robotic tonometry system, and a write model with an artificial skin and vessel. A transfer function model is developed using the results of the pulse testing and the mechanical characterization testing of the skin and vessel. To evaluate the model, the pulse waveforms are first reconstructed for various reference pulses using the model with tonometry data. They are then compared with pulse waveforms acquired by internal measurement (by the built-in pressure sensor in the vessel) the external measurement (the on-skin measurement by the robotic tonometry system). The results show that the model-produced pulse waveforms coinciding well with the internal pulse waveforms with small relative errors, indicating the effectiveness of the model in reproducing the actual pulse pressures inside the vessel.

## 1. Introduction

Arterial pulse pressure is an important biomarker for vascular diseases. In particular, being close to the heart, the pressure in aorta or the central aortic blood pressure (cBP) is strongly related to cardiovascular diseases, such as a heart attack. Thus, accurate measurements and assessments of cBP are critically important in predicting potential cardiovascular events and providing proper proactive medical treatments. Currently, the most accurate way to measure the aortic pressure for clinical reference is considered to be a direct blood pressure (BP) measurement method using an arterial catheter. For this invasive method, an arterial catheter is inserted into the artery of interest (such as the radial, dorsalis pedis, posterior tibial, and femoral arteries) to measure and monitor continuous BP and hemodynamics. While it is the most accurate, the invasive BP monitoring method with arterial catheters causes minor complications, such as local paresthesia, and carries the risk of serious complications, such as infection and nerve damage, particularly for children who have smaller arteries [1]. Thus, invasive BP monitoring methods are generally used in perioperative and intensive care medicine. Moreover, invasive BP monitoring demands an in-depth understanding of the BP measurement principle (such as insertion site, type of catheters, transducer readings) and the knowledge of BP waveforms to obtain correct and quality BP measurements, avoiding common pitfalls [2].

Alternative to the direct continuous BP measurement method with arterial catheters, various non-invasive BP monitoring methods are available. They include continuous or intermittent methods with their own advantages and disadvantages [2,3]. Intermittent non-invasive measurements using oscillometery are not as accurate as compared with those of an invasive method using an arterial line [4,5]. Ambulatory blood pressure monitoring (ABPMs) can measure intermittent BP, but they can be inconvenient and impractical for continuous monitoring of BP, particularly for critically ill patients, and cause discomfort during frequent measurements. More significant disadvantages of ABPMs are that the measurements can be susceptible to movements or positions of patients, and they cannot be used to derive the cBP waveform. Hence, the cuff-based method is limited to diagnosing or monitoring patients with hypertension.

Non-invasive radial artery applanation tonometry (AT) is suitable for the estimation of cBP waveforms produced from inverse transformed radial waveforms [6]. Radial artery AT is conducted by placing a portable tonometric device over the radial artery in the wrist and applying light pressure for flattening the artery. Due to the comfort and ease of use, various radial artery AT devices, including wearables, are being commercialized for continuous BP waveform monitoring. For example, BPro G2 is a wrist-worn band system that is capable of monitoring BP along with the heart rate and central aortic systolic pressure by applying a constant applanation force on the radial artery to capture the radial pressure waveforms [7]. Another example is the Bosimi Band, which can be placed on a patent’s wrist to monitor BP waveforms continuously and non-invasively [8]. The sensor built into the Bosimi Band uses a thin capacitive sensor that lightly touches the skin to capture a pulse wave signal that correlates with changes in arterial blood pressure.

Radial artery pulse waveforms provide useful clinical information related to cardiovascular risk factors [1,2,3,4]. Pulse waveforms contain various vascular indices, such as the augmentation index (AIx) [2,4,5], pulse wave velocity [6,7,8], and pulse pressure index (PPI) [9,10,11]. These are used to clinically estimate artificial stiffness and other vascular conditions. In addition, radial pulse waveforms are widely used clinically in oriental medicine. For example, the pulse diagnosis technique in oriental medical utilizes a variety of temporal and spatial features of the pulse waveforms such as pulse width, length, depth, and spectral power density in identifying a patient’s internal diseases or health status [12].

In recent years, with the advancement of wearable technologies, more radial artery AT devices are being developed for continuous, real-time, and non-invasive monitoring of vital signs. For wearable radial artery AT devices (such as smart watches and bands) or radial tonometers, the radial artery pulse pressure is recorded by a sensor that directly contacts and presses down on the skin. The collected data are then processed using sophisticated mathematical models/algorithms to derive or calculate the cBP waveforms [9]. Several studies demonstrated that mathematical relationships between the radial pulse pressure waveforms and those of the cBP can be developed in the form of transfer functions, and the transfer function can be used to predict the cBP [9,10,11]. Most of these transfer function studies involve human subject testing. In order to accurately estimate the cBP waveform from the radial artery AT, it is important to first develop a mathematical model for the relationship between radial intravascular blood pressure waveform and the measured radial pulse pressure by the sensor on the skin surface. Woo et al. mathematically modeled the skin mechanism based on the Kelvin–Voigt model to derive the radial intravascular blood pressure waveform from the pressure measured with the radial artery AT [12].

Despite the importance of obtaining radial intravascular pressure waveforms from radial artery tonometry, limited studies investigated the mathematical transfer modeling between the tonometry pulse data and the pulse pressure inside the radial artery. Perhaps, the need for clinical trials has hampered active investigations into the radial artery transfer function studies. While it is one of the most essential components for the clinical verification process, clinical trials involving human subject testing demands tremendous resources, cost, time, and efforts. Moreover, it can overlook person-to-person variations or human errors that can cause misleading information. In the case of radial pulse measurements, several factors can affect the radial pulse measurements (such as variation of skin properties among patients), and the accuracy of measurement is sensitive to patient variations, such as the posture of the patient. Thus, the needs exist for alternative methods that can perform radial pulse research, eliminating or minimizing human variations.

This research proposes to study a transfer function of radial pulse pressures using a robotic arterial tonometry measuring system and pulse generation system. The application of a pulse generation system capable of generating a radial pulse in an artificial wrist can significantly contribute to accurate transfer function modeling by offsetting various and unpredictable human factors. In addition to the need for mathematical modeling of arterial tonometry without human subject testing, the proposed research is anticipated to contribute to evaluation and validation of the accuracy, reliability, and usability of wearable sensors and wearable tonometry devices for clinical applications.

The primary goal of this study is to develop a mathematical model that relates the radial pulse pressure measured on the skin surface and the pressure inside the radial artery based on the measurements obtained by a robotic tonometry system. To this end, a test set up is created with three main components that include an artificial wrist model (consisting of synthetic skin and blood vessel), a pulse generation system, and a robotic tonometry system. Based on the cam mechanism, the radial artery pulse-generation system (RAPS) can reproduce in vivo radial pulse waveforms consistently and repeatedly so that it can act as a reference (or input) pulse pressure. In order to quantitatively measure the pulse pressure, the robotic tonometry system (RTS) is equipped with highly sensitive pulse sensors (an array of piezo sensors) and employs dexterous robotic manipulation technology to control the measuring direction and pressing force of the sensor with a laser guide. The RTS can automatically detect and compress the radial artery as well as collect the (output) pulse signals on the skin surface. Thus, RAPS and RTS systems can ensure the consistent testing conditions for pulse measurement, eliminating potential human-induced errors or variations. Using the test setup, a series of pulse tests are performed on the wrist model with various input pulse waveforms.

In addition to the pulsation testing, a separate DMA testing is conducted in order to characterize the mechanical properties of the skin and skin-vessel model. The results of the DMA testing are then used to develop a transfer function model along with the pulse data. This study intends to apply a modified Kelvin–Voigt model for estimating the radial skin dynamics caused by subcutaneous tissue, skin, and radial artery. The constants of the Kelvin–Voigt model are determined through an optimization algorithm based on the comparison of the signal measured on the skin with the robotic tonometry system (RTS) and the signal of the pressure sensor embedded in the vessel tube of the RAPS. In order to establish the transfer function that can be commonly applied to age-dependent waveforms with clinically different trends, the age-specific cams are designed based on the average waveforms of 10 s, 30 s, 60 s, and 80 s obtained through extensive clinical trials using RTS [13]. The age-specific cams are mounted on the RAPS to generate an age-dependent radial pulse pressure waveform, and the resulting waveforms are applied to the optimization algorithm to refine the constants of the Kelvin–Voigt model.

After developing the model, it is validated with age-related in vivo pulse inputs. For this verification study, the pulse waveforms that are reconstructed the model are compared and analyzed with those from the on-skin tonometry measurement data. The remaining major issue for the clinical application of the developed tonometry system is the ability to derive intravascular pressure waveforms using the tonometry results. Since the intravascular pressure waveforms are distorted by the stiffness and damping characteristics of skin layers and radial artery vessel [12], it is necessary to mathematically analyze the relationship between the internal pressures of the arterial vessel tube and the external pressures measured on the skin surface by tonometry method.

The next section provides a description of the configuration and operation principle of the RTS and the cam-based RAPS device. After describing the overall process of establishing the transfer function based on the comparison of the blood pressure waveform measured by built-in pressure sensor in the blood vessel tube and by RTS using a tonometry method, this paper presents the error analysis results between the waveform and the blood pressure waveform measured inside the blood vessel.

## 2. Proposed Modeling Process

The purpose of this section is to describe the proposed transfer function modeling method for this study. This section first presents a brief summary of the radial artery pulse-generation system and the robotic tonometry system. These are essential systems to obtain experimental data under consistent testing conditions for the modeling study. It then offers a description of the modeling process that is used in this study along with the experimental validation and analysis of the proposed model.

### 2.1. Radial Artery Pulse-Generation System (RAPS)

**Figure 1:**
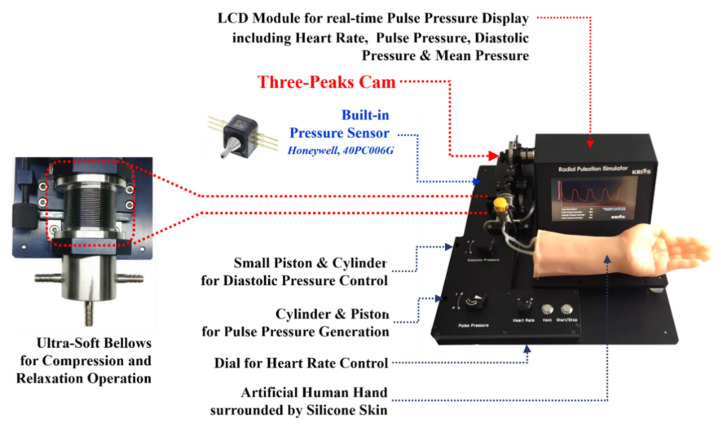
Developed three-peak cam-based radial pulsation simulator for generating age-dependent radial pulse waveforms.

Reproducing arterial pulse waveforms consistently and continuously is crucial to carrying out the proposed study. The authors developed a radial artery pulse-generation system or RAPS in a previous study [14]. Consisting of a cam manufactured with a pulse profile mounted on a DC motor and the cylinder/piston or cam follower module, the RAPS reciprocates according to the shape of the cam as the cam rotates to generate the prescribed radial pulse pressure waveform. For the current study, an enhanced radial pulse simulator prototype is employed as shown in Figure 1. In the new prototype, ultra-soft bellows were added to the cam follower module to compensate for the leakage and friction generated between the piston and cylinder in order to enhance the accuracy of the output radial pulse waveform. In addition to the bellows’ incorporation, a PID controller is incorporated to control the DC motor speed so that it can maintain a constant rotational speed regardless of the cam profile. This also enhanced the accuracy of the output waveform by eliminating the waveform distortion issue caused by the reduction of the DC motor speed at the peak points of the cam profile. Additional enhancements of the RAPS include adding a hall effect sensor capable of measuring the rotational speed of the DC motor to measure the heart rate and a small cylinder/piston module to adjust the diastolic blood pressure by regulating the pressure inside the tubing. The prototype is equipped with a small pressure sensor (Honeywell, 40PC006G) to monitor the internal pressure of tubing, representing an artery, in real time. This enhanced RAPS, along with cams manufactured with in vivo radial pulse data for different age groups, is used to provide a reference or input pulse pressure waveform to the wrist model.

### 2.2. Robotic Tonometry Pulse Measurement System

**Figure 2:**
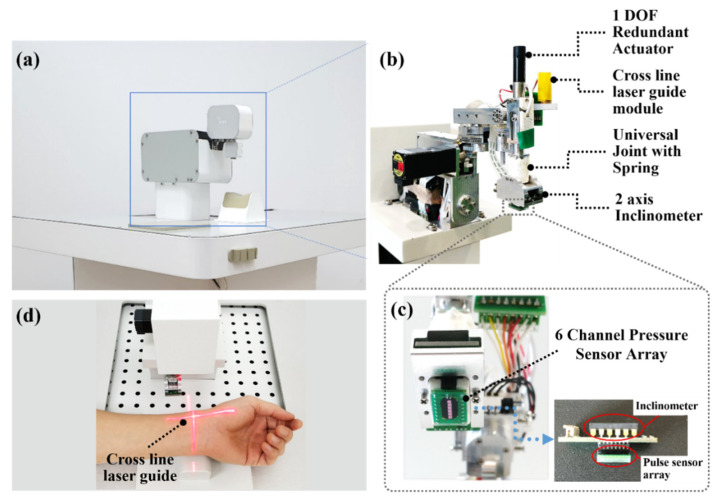
Robotic tonometry pulse measurement system to measure the precise pulse wave on the radial artery; (**a**) a picture of the robotic tonometry system, (**b**) components of a robotic pressure manipulator, (**c**) built-in piezo-resistive pressure sensor array, (**d**) demonstration of actual pulse measurement using a cross line laser guide.

In order to measure radial pulses without a human operator, this study will use a robotic tonometry pulse measurement system (RTS), which was developed in an earlier study by the authors. While the detailed design and performance of the RTS can be found in the study [15,16], this section intends to briefly recapture the working principle of the system and its key features as background information in the context of the current study. Figure 2a shows a photo of the RTS, which is a 5-axis motor system, allowing 3-degrees-of-freedom (DOF) linear translational motion and 2-DOF rotational motion. Thus, the pulse sensor module mounted on the end effector of the manipulator can be freely moved. As shown in Figure 2b, the RTS includes a pressure sensor module with an inclinometer, a spring-universal joint, and a 1-axis precision pressure motor. Figure 2c shows the details of the pressure sensor module, consisting of a piezo-resistive pressure sensor array. Piezo-resistive pressure sensors are one of the most prominent methods used to measure arterial pulse waves [17]. The 10 mm × 8 mm piezo-resistive pulse array was fabricated using an arrangement of 6 in-line sensor cells (C33, EPCOS, Munich, Germany). The in-line arrangement ensures a sufficiently large sensing area for pulse measurements. The surface of the pulse sensor is coated with silicone to prevent the sensor cells from fracturing by the contact force with the wrist skin [18]. The RTS employs a 2-axis inclinometer (SCA100T-D01, Murata, Manufacture Co., Shinjuku City, Japan) to measure the tilt of the pulse sensor relative to the skin surface along the gravitational axis. The universal joint with a spring is tilted in accordance with the tilting angle of the pulse sensor, and it can press the pulse sensor against the skin surface while maintaining constant posture and contact force. The sensor is capable of recording the pulse data at a sampling rate of 1000 Hz with a band pass filter from 0.1 to 27 Hz. The RTS uses a laser guide to identify the measurement position of the radial artery. Figure 2d shows a photo of an in vivo testing for measuring a radial arterial pulse using the RTS. Note that a cross line laser was used to guide the pulse wave measurement position on the subject’s wrist.

### 2.3. Proposed Transfer Function Modeling Process

**Figure 3:**
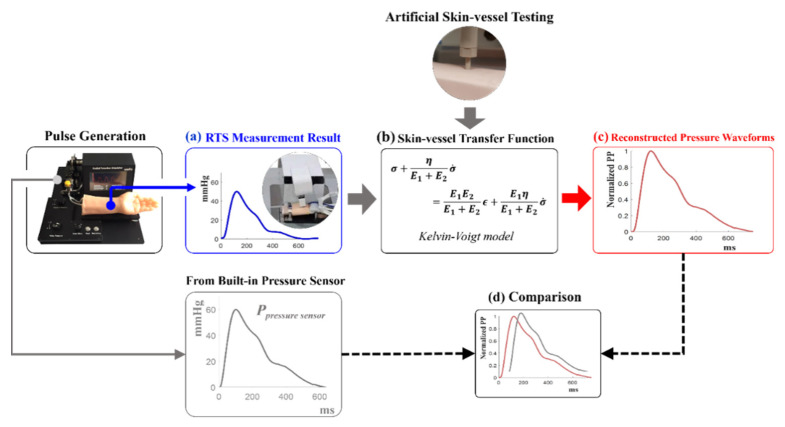
A flow of the overall process: (**a**) Pulse pressure waveform measured on skin above the simulator’s wrist using RTS, (**b**) skin-vessel transfer function modeling based on artificial skin and vessel mechanical testing results, (**c**) intravascular pulse pressure waveforms reconstructed from the RTS measurement results by the skin-vessel transfer function, (**d**) the comparison of the differences between the reconstructed waveforms and the pulse pressure in vessel measured by pressure sensor.

This section explains the whole process of establishing a transfer function that can accurately predict the blood pressure waveform inside the radial artery from the pulse pressure waveform measured on the skin above the simulator’s wrist by using the RTS. Figure 3 offers a visual illustration of the proposed study. As shown in Figure 3, the cam-based RAPS generates a reference radial pulse waveform. In order to generate an age-dependent radial pulse pressure waveform in the cam-based RAPS, the fabricated 15, 35, 65, and 85-year-old cams were mounted on the RAPS, respectively. It is important to note that, at the same time, the blood pressure waveform inside the blood vessel was measured using a small blood pressure sensor (Honeywell, 40PC006G) built into the simulator. This internal pulse pressure will be compared with that reconstructed by the model developed in this study. The generated waveform for each age was measured on the skin above the simulator’s wrist using RTS as shown in Figure 3a. The measured data will then be used to develop a skin-vessel transfer function model along with mechanical property data of the vessel and skin-vessel model obtained by a separate characterization testing of the skin/vessel sample as shown in Figure 3b. The transfer function model with the embedded skin-vessel property information can reconstruct the internal radial pulse pressure waveform. In other words, using the RTS measurement waveforms, the model can estimate the radial pulse pressure inside the vessel (tubing). In order to evaluate the performance of the model, different age-related radial pulse inputs will be used. Again, these input/reference pulse pressures inside the vessel are directly measured using a pressure sensor installed in the RAPS. They will be compared with the reconstructed radial pulse waveforms by the model for analysis.

## 3. Experimental Evaluation of the Skin-Vessel Model

In order to develop a transfer function model for the internal pressure in the artery and the externally measured tonometry pressure, the properties of the skin and the vessel need to be characterized. To determine the mechanical properties of the artificial skin and vessel, this study preformed compression testing using a dynamic mechanical analyze or DMA (RSA3, TA Instruments). The DMA ensures the purest mechanical data through independent control of deformation and measurement of stress by using separate motors and transducers.

**Figure 4:**
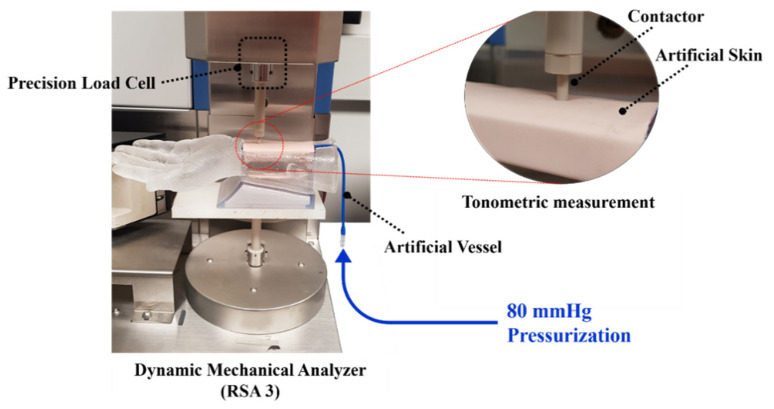
Experimental setup for compression testing of the artificial wrist model.

Figure 4 shows the experimental set up where the artificial wrist model with the artificial vessel and skin is installed in the DMA. In order to simulate the diastolic pressure in the vessel, the pneumatic pressure of 80 mmHg is filled in the artificial vessel in the wrist model. The indenter mounted on the upper fixture of the DMA compresses the wrist model to measure the mechanical properties of the artificial vessel and skin. The indenter with the tip diameter of 4 mm, which is similar to the outer diameter of the vessel, compresses the skin/vessel by 1 mm at the speed of 1 mm/s. Compression of the indenter was performed with an indentation depth of 1 mm similar to the human radial artery tonometric measurement conditions and a speed of 1 mm/s similar to human pulsation of 60–100 bpm (beats-per-minute). The DMA then records the force and displacement with the precise load cell and transducer. Compression tests were performed on the vessel alone and the skin-vessel combined. Compression tests were performed on the skin–vessel combination, not skin alone, to accurately reflect the physical condition that occurs when soft silicone-based skin with viscoelastic mechanical properties is combined with blood vessels. In both of these tests, the vessel is pressured at the diastolic pressure of 80 mmHg.

**Figure 5:**
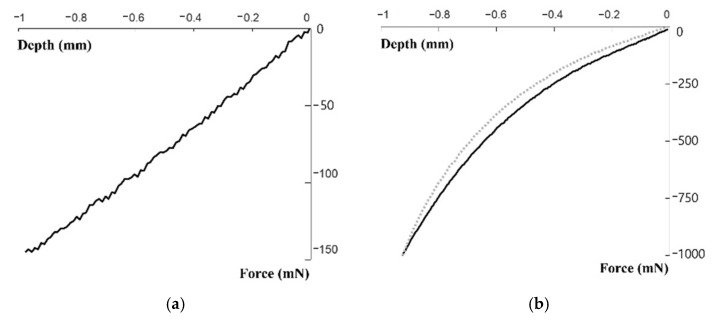
Experimental results of the artificial wrist’s mechanical properties: (**a**) Artificial blood vessel filled with 80 mmHg pneumatic pressure, (**b**) artificial blood vessel filled with 80 mmHg pneumatic pressure and wrapped with the skin layer.

Figure 5 shows the compression testing results for the artificial vessel only (Figure 5a) and the vessel with the skin layer (Figure 5b). As shown in Figure 5a, the force increases almost linearly as the compression depth increases, reaching up to 150 mN at the depth of 1 mm. By converting the force–displacement relationship to the stress–strain relationship and taking the slope of the graph, the modulus of elasticity of the vessel with the diastolic pressure is calculated to be 39.58 MPa. Figure 5b shows the results of the second testing that was conducted with the presence of a skin attached to the vessel. As shown in the figure, the vessel with the skin layer exhibits a nonlinear and hysteresis behavior. During the loading cycle (the solid line), as the compression depth increases to 1 mm, the force increases nonlinearly to 1000 mN, which is much higher than compared with the vessel only case. For the unloading cycle (a dotted line), as the compression depth decreases from 1 mm to zero or the original position, the force also decreases but the magnitude is smaller at a given depth when compared to the loading case. This hysteresis behavior is due to the viscoelastic properties of the added skin layer. To extract the mechanical properties of the skin layer from the compression testing, an optimization study is used, which will be described in the next section along with the transfer function modeling method.

## 4. Modeling of Skin-Vessel Transfer Function

This part of the study intends to construct a mathematical model that combines the elasticity of the radial artery and the physical properties of the skin in order to estimate the waveform in the blood vessel using a blood pressure waveform measured externally by a tonometry method. To construct a transfer function model capable of reconstructing intravascular pressure, in this section, the radial artery and skin of the wrist were modeled using the Kelvin–Voigt model with an additional serial spring in the parallel structure of a spring and a dashpot. Next, the mechanical properties of artificial skin were extracted through optimization based on the overall stress–strain measurement results using DMA. A model capable of regenerating intravascular pressure waveforms was then completed by applying the determined physical property values to the Kelvin–Voigt model.

### 4.1. Modeling of Artificial Skin and Blood Vessel

Human skins exhibit complex physical behaviors, and many different models have been studied to capture them. One of the common mathematical methods that is used to model human skin behaviors is to use the Kelvin–Voigt model, in which Hookean springs (elastic elements) and Newtonian dashpots (viscous elements) are connected in parallel [19,20,21]. Recently, Sung Hun Woo et al. mathematically modeled radial skin dynamics caused by the subcutaneous tissue, skin, and radial artery using a modified Kelvin–Voigt model that describes the bulk property of collagen fibers and the arterial vessel [12]. The modified model was able to properly estimate the exact absolute blood pressure inside the radial artery using a watch-type tonometry device on the skin surface. However, the existing method has a limitation in that it is not possible to regenerate the blood pressure waveform inside the radial artery, which contains important clinical information of the cardiovascular system such as the arterial stiffness and pulse wave velocity, by using the measurement results of the tonometry method.

**Figure 6:**
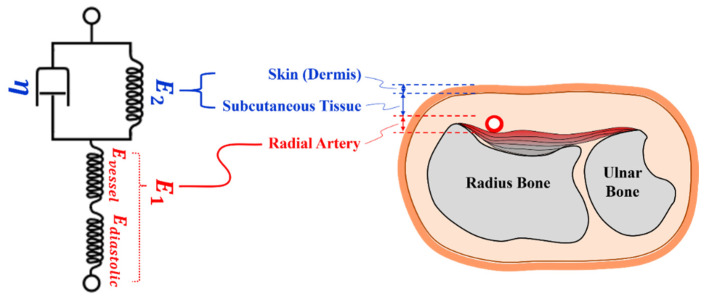
An illustration of spring-dashpot based Kelvin–Voigt model for human wrist.

The current study aims at developing a transfer function model that is capable of reconstructing the internal pulse waveform using the tonometry data. The model can be used to estimate the arterial pressure in the vessel. Moreover, the reconstructed pulse waveforms can be used to obtain clinically meaningful information, such as an augmentation index. The augmentation index is often used to indirectly measure vascular stiffness. To attain the goal, this study employs a two-layer Kelvin–Voigt model and an optimization algorithm that determines the spring element ($E_{2}$) and a damping element (η) for the skin model from the DMA testing data. Figure 6 illustrates the model and the cross-section of the wrist. As shown in Figure 6, the first layer where two spring elements are arranged in a series configuration represents the stress and the strain relationship of the vessel. The elastic modulus of the vessel is affected by the intravascular pressure of the vessel (diastolic pressure, $E_{diastolic}$) and the elasticity of the vessel itself ($E_{vessel}$). Since the diastolic pressure is fixed at 80 mmHg, we can assume that the modulus of elasticity of the vessel of the first layer ($E_{1}$) is primarily affected by the stress–strain relationship of the vessel, which was determined as 39.58 MPa by the DMA testing (see Figure 5a). The second layer in the model describes the relationship between the stress and the deformation of the skin. As shown in Figure 6, a parallel configuration of a spring element ($E_{2}$) and a damping element (η) is used to represent the “skin” layer, exhibiting a viscoelastic nature of the property. The viscosity parameter, *η*, and the elastic modulus parameter, $E_{2},$ are obtained from the skin-vessel testing.

The two-layer Kelvin–Voigt model for the skin and the vessel discussed above can be expressed as a differential equation (see Equation (1)). In the equation, σ is stress and ϵ is strain. The values of the parameters $E_{1}$, $E_{2}$, and η need to be determined experimentally to solve the equation.

(1) $$\sigma +\frac{\eta }{E_{1}+E_{2}}\dot{\sigma }=\frac{E_{1}E_{2}}{E_{1}+E_{2}}\epsilon +\frac{E_{1}\eta }{E_{1}+E_{2}}\dot{\epsilon }.$$

### 4.2. Skin Parameter Determination Based on Optimization Study

To determine skin parameters, the modulus, $E_{2}$, and the viscosity, η, from the skin-vessel combined data obtained by DMA testing as described in Section 3, the optimization study was performed. First, Equation (1) was rearranged for the strain dependent on the parameters $E_{2}$ and η, and an explicit solution of the strain was obtained. This is because the parameters $E_{2}$ and η can be determined through an optimization study that minimizes the error between the reconstructed strain, $\hat{\epsilon }$, and the measured strain, ϵ.

Through dividing Equation (1) by $E_{1}\eta /\left(E_{1}+E_{2}\right)$ and considering the strain variable as unknown, one can obtain the first order differential equation with right-hand side terms determined by the measured data of stress variables.

(2) $$\dot{\epsilon }+\frac{E_{2}}{\eta }\epsilon =\frac{E_{1}+E_{2}}{E_{1}\eta }\left(\sigma +\frac{\eta }{E_{1}+E_{2}}\dot{\sigma }\right).$$

One observes that as the damping parameter (η) decreases, the proportion of the first derivative of stress on the right-hand side in Equation (2) becomes smaller, which means that the strain variable follows the change of the stress variable immediately. On the other hand, a large value for the damping parameter means that the first derivative of stress has a large affect on (2), which leads to the inconsistency in the shape of waveforms of stress variables and that of the strain variable. Before solving (2), we introduce some notations for convenience’s sake:

$$a=\frac{E_{2}}{\eta },\;F=\frac{E_{1}+E_{2}}{E_{1}\eta }\left(\sigma +\frac{\eta }{E_{1}+E_{2}}\dot{\sigma }\right).$$

Here, the periodic nature of the waveforms must be mentioned in order for the problem to be defined. Since the waveform is generated by a mechanism that follows the cam running at the same rotational speed, say ω, the pressure waveform in the tube is periodic with a period T=2π/ω. In summary, we can rewrite (2) as follows:

(3) $$\dot{\epsilon }+a\epsilon =F,\;\mathrm{on}\;\left(0,T\right).$$

By the general solution of the first order differential equation, the reconstructed strain is obtained as

(4) $$\hat{\epsilon }\left(t\right)=\exp\left(-at\right)\left(\int_{0}^{t}\exp\left(a\tau \right)F\left(\tau \right)d\tau +C\right)$$

where C is a integration-constant determined by some boundary condition. Since the magnitude of strain vanishes at the end of the releasing experiment (see Figure 5b), the boundary condition below is imposed:

(5) $$\hat{\epsilon }\left(T\right)=0.$$

Using (5), one can determine C in (4) as

(6) $$C=-\int_{0}^{T}\exp\left(a\tau \right)F\left(\tau \right)d\tau .$$

Thus, by substituting the value of *C* calculated by (6) to Equation (4), we can determine reconstructed deformation, ${\hat{\epsilon }}_{E_{2},\eta }$, explicitly, which depends on the parameters $E_{2}$ and η. The parameters $E_{2}$ and η can be obtained through an optimization study that minimizes the error between the reconstructed strain, ${\hat{\epsilon }}_{E_{2},\eta }$, and the measured strain, ϵ. Hence, an objective function, Equation (7), is defined as $L^{2}$-difference of reconstructed strain and the measured strain, i.e.,

(7) $$Obj_{E_{2},\eta }=\sqrt{\int_{\left[0,T\right]}^{}{\left({\hat{\epsilon }}_{E_{2},\eta }-\epsilon \right)}^{2}\mathrm{ds}}.$$

**Figure 7:**
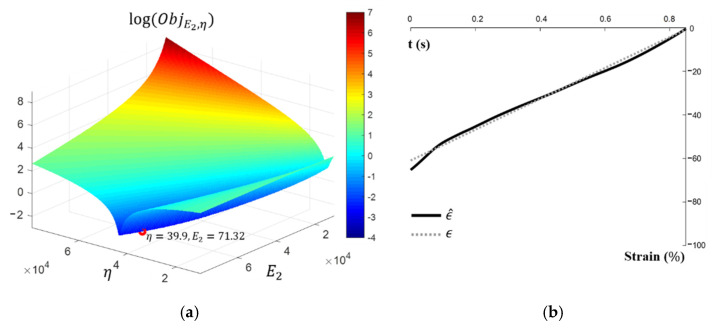
Results of the optimization study (**a**) Change of objective function, $Obj_{E_{2},\eta }$, with respect to $E_{2}$ and η (left), whose value is minimized when $E_{2}=71.32\;\mathrm{MPa}$ and η=39.94 MPa s, (**b**) The comparison between the reconstructed strain and deformation.

When the objective function is small enough for particular parameters, it means that the reconstructed waveform generated by the corresponding parameter is similar to the targeted waveform. Thus, parameters $E_{2}$ and η are determined so that they minimize the objective function. Figure 7a shows the change of an objective function with respect to $\left(E_{2},\eta \right)$ in Ω≔(0.80 MPa)×(0.80 MPas). The objective function, $Obj_{E_{2},\eta }$, is evaluated on 222 × 222 pairs of $\left(E_{2},\eta \right)$, which are located on the nodes of the grids generated by dividing Ω with a uniform mesh-size h=80/221. Comparing the values of $Obj_{E_{2},\eta }$, the parameters are determined as $E_{2}=71.32\;\mathrm{MPa}$ and η=39.94 MPa s. The reconstructed strain obtained by solving (1) with parameters chosen by the minimizing objective function process is compared with real strain in Figure 7b. We see that the reconstructed strain matches the real strain.

### 4.3. Reconstruction of the Pulse Pressure Waveform in Artificial Vessel

Analyzing how the waveform changes as it passes through the viscoelasticity material, a method to reconstruct the waveform of pulse pressure in an artificial vessel from the waveform measured on the skin is proposed. The process is based on the Kelvin–Voigt model together with the concept that a change of strain is proportional to the change of pressure.

The process of reconstructing the strain variable from the measured stress on the skin is similar to that described in Section 4.2. First, by solving the Kelvin–Voigt model, one obtains Equation (4) as an expression for a reconstructed strain. However, to determine a constant *C* in Equation (4), a different boundary condition must be used. Since a waveform is periodic with a period T, we shall impose periodic-type boundary condition, i.e.,

(8) $$\hat{\epsilon }\left(0\right)=\hat{\epsilon }\left(T\right).$$

By the above condition, the constant C in Equation (4) is determined as

(9) $$C=\frac{\exp\left(-aT\right)\int_{0}^{T}\exp\left(a\tau \right)F\left(\tau \right)d\tau }{1-\exp\left(-aT\right)}$$

In summary, the explicit equation for the reconstructed strain of the artificial vessel is obtained as

(10) $$\hat{\epsilon }\left(t\right)=\exp\left(-at\right)\left(\int_{0}^{t}\exp\left(a\tau \right)F\left(\tau \right)d\tau +\frac{\exp\left(-aT\right)\int_{0}^{T}\exp\left(a\tau \right)F\left(\tau \right)d\tau }{1-\exp\left(-aT\right)}\right).$$

To reconstruct the waveforms of pressure inside the artificial vessel based on the strain variable obtained above, the relationships between the pressure inside the vessel and the strain of the tube was derived from the collapsible tube modeling in [22] where the changes of pressure are assumed to be proportional to that of the square root of area, i.e.,

(11) $$p\left(t\right)=p_{0}+\beta \left(\sqrt{A\left(t\right)}-\sqrt{A_{0}}\right)$$

where β is some coefficient determined by the vessel’s thickness, cross-sectional area, Young’s modulus, and Poisson ratio. Based on Equation (11) and the fact that $A=\pi r^{2}$ where *r* is the radius of the vessel, one can derive the relation that

(12) $$p\left(t\right)-p_{0}=\beta \sqrt{2\pi }\;\left(r\left(t\right)-r_{0}\right)$$

Finally, noticing that $\hat{\epsilon }\left(t\right)=\left(r\left(t\right)-r_{0}\right)/2r_{0},$ we conclude that the pulse pressure waveforms, Equation (13), are proportional to waveforms of ϵ obtained by Equations (5) and (7), i.e.,

(13) $$\begin{array}{ll} p\left(t\right) & =2r_{0}\beta \sqrt{2\pi }\;\hat{\epsilon }\left(t\right)+p_{0}\\ & =2r_{0}\beta \sqrt{2\pi }\left(\int_{0}^{t}\exp\left(a\tau \right)F\left(\tau \right)d\tau +\frac{\exp\left(-aT\right)\int_{0}^{T}\exp\left(a\tau \right)T\left(\tau \right)d\tau }{1-\exp\left(-aT\right)}\right)+p_{0} \end{array}$$

## 5. Performance Evaluation of the Model and Analysis

The goal of this section is to evaluate the performance of the skin-vessel transfer function model developed in the previous section. To this end, a set of age-related radial pulse waveforms are used as reference waveforms, and they are compared with the pulse waveforms that are reconstructed by the transfer function model using tonometry data. In addition to the different pulse waveforms, pulse rates are changed to further evaluate the performance and the reliability of the model. To quantitatively evaluate the performance, the error analysis is conducted between the model generated pulse waveforms and those measured by the built-in pressure sensor for the reference pulse waveforms. Moreover, the augmentation index is compared between the sensor measured pulses waveforms and the reconstructed pulse waveforms.

**Figure 8:**
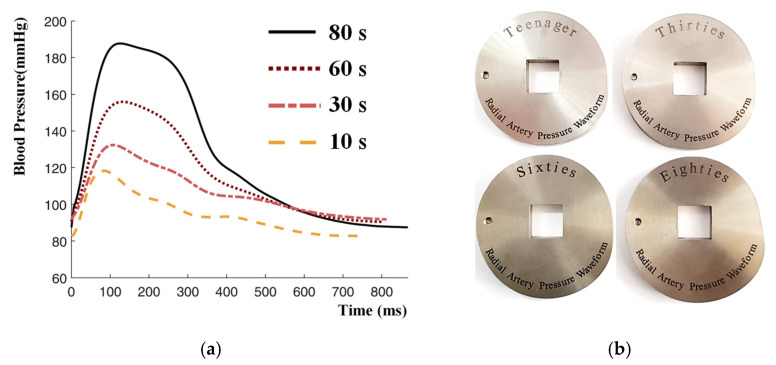
Experimental setup using age-dependent cams; (**a**) tonometric measurement results of radial artery pressure waveforms of subjects by age, (**b**) fabricated age-dependent cams.

To generate age-dependent radial pulse pressure waveforms using the cam-based RAPS, the study uses the cams designed and fabricated based on in vivo data in an earlier study by the authors. While details of the human subject testing and cam designs can be found in [13,14], this study has included Figure 8 in order to show the in vivo radial pulse waveforms for the selected age groups (10 s, 30 s, 60 s, and 80 s) and the fabricated cams. Each of these cams is mounted in the RAPS to generate the average waveforms for the four different age groups as reference waveforms. As explained in Section 2.3, the robotic tonometry system is then used to measure the external pulse waveforms on the skin surface of the wrist model. To evaluate the consistency of the sample waveform data generated from the RAPS, the averaged in vivo waveform and each sample’s data were compared. The averaged in vivo waveform for age groups was obtained from human subject testing by averaging the 40 pulse waveforms of each age group recorded in the steady-state region of the measured radial pulse waveforms [14]. The error between sample waveforms ($u_{i}^{’},\;i=1,\ldots ,40)$ and the averaged waveform (u) was calculated by using the following equation. The error was less than 5% for all age groups.

(14) $$\frac{\frac{1}{40}\sqrt{\sum_{i=1,\ldots ,40}\int {\left(u_{i}-u\right)}^{2}\;\mathrm{ds}}}{\sqrt{\int u^{2}\mathrm{ds}}}$$

**Figure 9:**
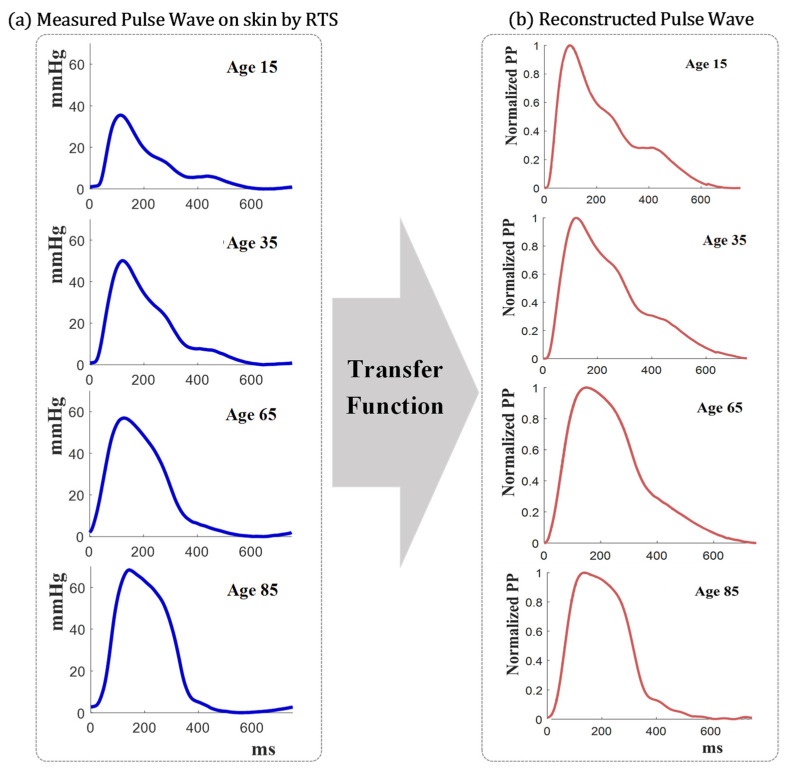
Reconstruction of age-related intravascular pressure waveforms: (**a**) Pulse pressure measured on the skin by the RTS, (**b**) reconstructed intravascular pulse pressure waveforms by the skin-vessel transfer function.

Figure 9a shows “external” radial pressure pulse waveforms measured by the RTS for different age groups. The transfer function model used these waveforms to estimate and reconstruct the “internal” radial pulse waveforms. Figure 9b shows the reconstructed pulse waveforms that are normalized for the purpose of comparison and analysis.

**Figure 10:**
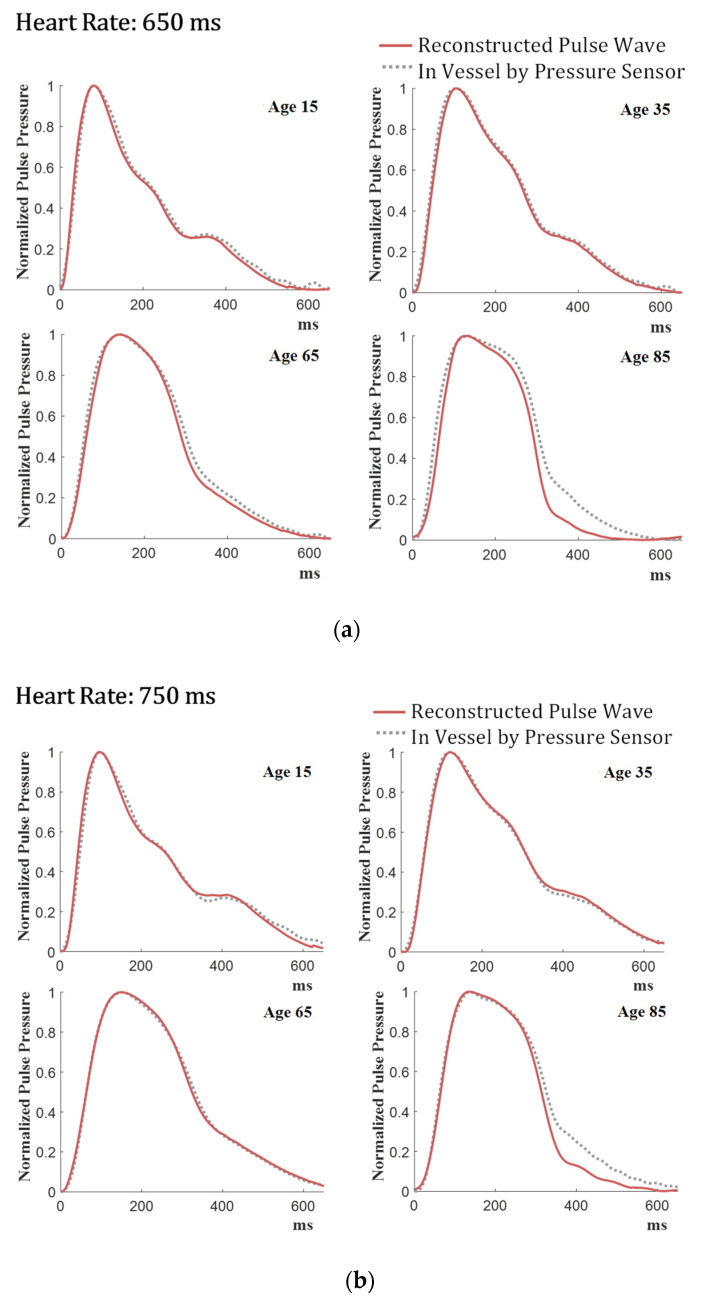
Comparison between the normalized reconstructed pulse waveforms and intravascular waveforms: (**a**) 650 ms heart rate, (**b**) 750 ms heart rate.

Figure 10 compares the pressure sensor-measured pulse waveforms and the model-generated pulse waveforms after normalizing them. Two separate rotational speeds of the cam are used to evaluate the robustness of the model to phase changes in the pulse waves due to different “heart rates.” Figure 10a,b shows overlaid plots of pulse waveforms for the period of the waveform 650 ms and 750 ms, respectively. As shown in Figure 10, reconstructed pulse pressure waveforms coincide well with the internal pulse pressure waveforms for all age groups considered in this study, particularly the peaks of the waveforms. While the 80 s results show some discrepancies in the tail end of the waveform at the late stoke of the pulse, they are not unacceptable as the peaks of the waveforms bear more important clinical connotation in arterial pressure measurements.

To analyze the results quantitatively, relative least square error (LSE or $L^{2}$-error) and relative augmentation index (AI) are evaluated. First, the error was analyzed by applying the least square error (LSE) method, which effectively calculates the total error by summing all the errors between the predicted value and the actual value. To calculate this error, Equation (15) below was derived by introducing the reconstructed pulse waveforms, $\hat{P}$, and intravascular waveforms, P, measured with the built-in pressure sensor.

(15) $$L^{2}-\mathrm{error}=\frac{\int_{T}{\left(\hat{P}-P\right)}^{2}\mathrm{dx}}{\int_{T}{\left(P\right)}^{2}\mathrm{dx}}$$

Table 1 shows the $L^{2}$-error for the periods of 650 ms and 750 ms and all age groups considered. As shown in the table, the relative $L^{2}$-errors are less than 10% for three age groups (15, 35, and 65 years). For the oldest age group (85 years), the error is over 10% for both heart rates. The small $L^{2}$-errors around 10% results indicate that the reconstructed pulse waveforms closely match with that of the intravascular pulse waveforms.

To further analyze the results, we computed relative *L^2^*-errors between measured waveforms on skin and intravascular waveforms (see Table 2). As shown in the table, the relative *L^2^*-errors range from 16.65% to 19.35%. As compared with the relative *L^2^*-error data between the reconstrued waveforms and intravascular pressure waveforms in Table 1, the *L^2^*-errors in Table 2 are significantly high. Further comparing Table 1 and Table 2, the difference in errors is as high as 16%, and the overall errors are reduced by 11.58% on average. This analysis indicates that the mathematical model developed in this study more accurately represents the intravascular pulse pressures as compared with the actual measured pulse pressure using a tonometer. The result further implies that the developed model can effectively estimate the internal pulse pressures by using the on-skin tonometry measurements.

Beyond the mathematical error analysis, it is important to analyze how the reconstructed waveform captures physiological information of arterial pulses. Thus, this study uses the augmentation index (AI) as another measure for the evaluation of reconstructed waveforms. AI is one of the frequently used parameters for vascular aging [23,24]. The AI of the pulse pressure, P, (AI_P_) is defined as the ratio of the late systolic pressure of P to the early systolic pressure of P or the ratio of the second peak of the pulse waveform to the first peak of the waveform. Table 3 shows a summary of the relative AI errors, which are the differences of AI_P_ between the reconstructed pulse waveforms and the intravascular pulse waveforms. As shown in Table 3, the relative AI errors are lower than 6.22% for all cases, indicating that the reconstructed waveforms by the model can capture the systolic pressures of the actual pulses inside the vessel.

**Figure 11:**
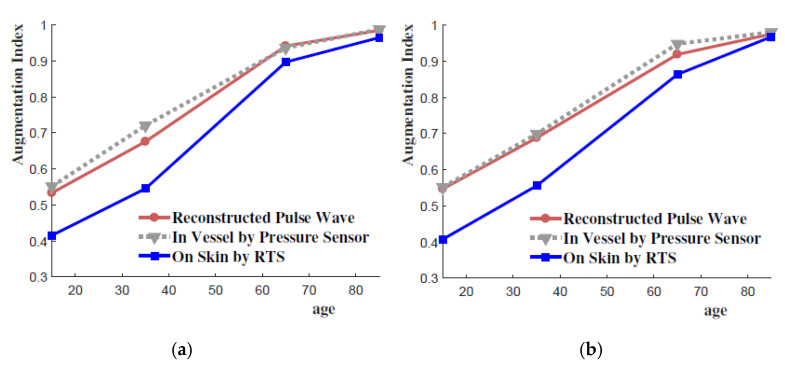
Comparison of AI of pulse pressure waveforms: (**a**) 650 ms heart rate, (**b**) 750 ms heart rate.

Figure 11 further compares the AIs of three pulse waveforms: (1) reconstructed pulse waveforms by the transfer function model, (2) intravascular pulse waveforms (or “internal” pulse waveforms) that are measured by the built-in pressure sensor, and (3) pulse waveforms on the skin surface (or “external” pulse waveforms) that are measured by RTS. As shown in the figure, there are substantial differences of AI values between the internal pulse waveforms and the external pulse waveforms due to the effect of the skin and vessel properties. The figure shows that the AI of the model-generated pulse waveforms closely match with that of the internal pulse waveforms. This result indicates that the discrepancies of AIs between the internal and external pulse waveforms are reduced by the reconstruction process by the Kelvin–Voigt model based on the skin and vessel properties. The result further indicates that the developed model can effectively estimate the internal pulse pressures by using the on-skin tonometry measurements, demonstrating that it acts as a transfer function that relates the external and internal pulse pressures.

## 6. Conclusions

This study has presented a skin-vessel transfer function model that is capable of estimating the blood pulse pressure inside a radial artery and reconstructing arterial pulse pressure waveforms using the tonometry data. As described earlier, most transfer function studies involve human subject testing. On one hand, they are immensely valuable because clinical trials involving human subjects can offer more direct and significant clinical reference results. On the other hand, human subject testing can be less desirable as it can be resource intensive. Moreover, it is subject to human errors originated from various factors (such as, person-to-person variations in physical dimensions and properties of skin and operator-to-operator variations in pulse measurement methods). Thus, the transfer function research involving human subject testing may not be suitable for investigating how the test subject’s variations (for example, in mechanical properties of skin and arteries) affect the accuracy of pulse pressure estimations. Not accounting for such realistic variations can result in misleading information when it comes to developing transfer function relationships between the internal and external pressures. The current work, therefore, has focused on developing a transfer function model accounting for the mechanical properties of skin and blood vessel. In developing such a model, it is important to have consistent input and output data without variations. To this end, the current study has employed a radial pulse generator (RAPS) to produce reference radial pulse waveforms that contain in vivo radial pulse characteristics. The reference pulses were measured on the surface of an artificial skin using a robotic tonometry pulse measurement system (RTS). The RAPS and RTS systems ensured consistent and repeatable results without involving human subjects and operators, eliminating man-made errors or uncertainties.

This study adopted a modified Kelvin–Voigt model in order to reconstruct internal pulse pressures using the RTS data. After performing a series of characterization testing on the skin-vessel model using a dynamic mechanical analyzer, the results of the characterization testing along with an optimization algorithm were used to build the transfer function model. To evaluate the performance of the model, this study compared and analyzed the pulse waveforms by the model and those of measured by the embedded pressure sensor for four different age groups’ average pulse waveforms with two different rotational speeds. The results show that the relative *L^2^*-errors between the reconstructed pulse waveforms by the model and the intravascular pulse waveforms are smaller than those for the measured and the intravascular pulse waveforms, indicating that the reconstrued pulse waveforms can more accurately represent the intravascular pulse waveforms as compared with the measured pulse waveforms. For the cases considered in this study, the relative *L^2^*-error between the on-skin measurement and the intravascular pulse waveforms are reduced by 11.58% on average with the transfer function model developed, demonstrating the effectiveness of the model in estimating intravascular pulse pressures. For further physiological analysis, the augmentation index (AI) was employed, which is a commonly used measure for vascular aging and arterial stiffness. The relative AI error analysis between the pulse waveforms obtained using the tonometry method and those obtained inside the vessel exhibit noticeable discrepancies. This is because the properties of the skin and the vessel distort the internal pulse pressures as they travel through them and are measured on the surface of the skin. The results further show that the relative AI error between the model-generated pulse waveforms and the internal ones are small (about 6% to less than 1%), indicating that the model can compensate the effect of the skin-vessel and effectively capture the AI of the internal pulse waveforms. This study has demonstrated the effectiveness of the transfer function model in producing the pulse waveforms that are close to the actual internal pulse waveforms. In particular, the model can reconstruct the internal pulse pressure waveforms so that they can be used for more meaningful analysis of the waveforms. Thus, the model is anticipated to apply to non-invasive radial pulse pressure measurements to monitor the “true” arterial pressure using the tonometry sensor measurements.

In future study, the methodology used in this study for the development of transfer function models is further anticipated to expand by incorporating the variation of the skin properties and a wide range of pulse frequencies or heart beats. Various artificial wrist models for each skin and blood vessel characteristic of a person will be created and evaluated using the methodology developed in this study with an aim to infer the intravascular pressure of a real person in a non-invasive way. To this end, a library of skin data considering various parameters, such as gender, age, and body mass index (BMI) will be created, and an enhanced transfer function model using the library of data will be developed. Moreover, since the proposed transfer function method with the robotic tonometry system could reconstruct pulse waveforms in real time after measuring pulses remotely, future research will explore its application for emerging healthcare technologies, such as remote patient monitoring based on Internet of Things [25,26]. These will contribute to the advancement of wearable healthcare technology with calibrations of wearable sensors and performance evaluations and validations of arterial tonometers.

## Figures and Tables

**Table 1:** Relative *L*^2^-errors of reconstructed pulse wave.

Age Group (Year)	650 ms	750 ms
15	5.51%	6.08%
35	4.16%	2.68%
65	6.41%	2.19%
85	14.87%	11.29%

**Table 2:** Relative *L*^2^-errors between intravascular waveforms in vessel and waveforms measured on skin.

Age Group (Year)	650 ms	750 ms
15	18.17%	18.91%
35	18.62%	18.35%
65	18.85%	16.92%
85	19.35%	16.65%

**Table 3:** Relative AI errors of reconstructed pulse wave.

Age Group (Year)	650 ms	750 ms
15	3.30%	0.85%
35	6.22%	1.52%
65	0.54%	3.08%
85	1.63%	0.52%

## Data Availability

Not applicable.
